# Editorial: Novel approaches for sustainable crop yield and management of plant-parasitic nematodes

**DOI:** 10.3389/fpls.2023.1274757

**Published:** 2023-08-29

**Authors:** Maria L. Inácio, Jorge M. S. Faria, Solveig Haukeland

**Affiliations:** ^1^ INIAV, I.P., National Institute for Agrarian and Veterinary Research, Oeiras, Portugal; ^2^ GREEN-IT Bioresources for Sustainability, Instituto de Tecnologia Química e Biológica, Universidade Nova de Lisboa (ITQB NOVA), Oeiras, Portugal; ^3^ MED, Mediterranean Institute for Agriculture, Environment and Development & CHANGE—Global Change and Sustainability Institute, Institute for Advanced Studies and Research, Évora University, Évora, Portugal; ^4^ Plant Health Theme, ICIPE, International Centre of Insect Physiology and Ecology, Nairobi, Kenya; ^5^ Division of Biotechnology and Plant Health, NIBIO, Norwegian Institute for Bioeconomy Research, Ås, Norway

**Keywords:** biocontrol, diagnostic, *Globodera* sp., *Meloidogyne* sp., plant engineering, plant-parasitic nematodes, *Pratylenchus* sp., resistance

Plant-parasitic nematodes (PPNs) are a major concern in agriculture as they cause significant crop damage resulting in yield losses and economic losses for farmers (FAO, 2019). For the past 50 years, the control of PPNs has relied heavily on the use of synthetic nematicides and soil fumigants, which have been effective in rapidly controlling nematode populations. However, due to environmental and health concerns, many traditional nematicides have been banned or withdrawn from the market. To achieve sustainable PPN control, it is advisable to adopt control strategies that are safer and more selective. These strategies include the use of bionematicides, biocontrol agents, cultural methods, and plant resistance (Jones et al., 2013). Bionematicides include biochemical antagonists such as natural products or microbial antagonists, which can induce chemical and/or physical damage against PPNs. Biocontrol agents, such as beneficial nematodes, fungi, and bacteria, can then be used to control PPNs (Pires et al., 2022). Cultural methods, such as crop rotation and the use of resistant cultivars, can also play a significant role in PPN control. The use of resistant cultivars is another effective strategy, as it involves breeding plants with resistance genes that can withstand PPN infections (Djian-Caporalino et al., 2014).

The aim of this Research Topic on research for integrated management of plant parasitic nematodes is to explore and advance innovative strategies for effectively control of these pests in a sustainable manner. This includes improving identification methods, investigating the potential of biological control agents and developing functional genomics for targeted control. By addressing these research areas, scientists can contribute to the development of sustainable and effective management strategies for plant parasitic nematodes.

The contributions include new updates in the identification and distribution of the impactful root-knot nematodes (RKNs) (Rusinque et al.), the use of alternative products to control different PPNs (Elsharkawy et al.; Pulavarty et al.; Kim et al.) and the investigation of genomic tools to confer disease resistance (Joshi et al.; Westerdahl et al.).

The accurate diagnostic of diseases caused by PPNs is the first step for preventing the spread of these pests, being essential for the sustainable management of cultural systems (Palomares-Rius et al., 2021). This is even more important because different nematode species can have different host ranges and pathogenicity. Rusinque el al. conducted extensive surveys across Portugal for the detection of RKNs, in close collaboration with phytosanitary authorities, confirming other reports on the global distribution of *Meloidogyne* sp. This study included crops of economic significance grown intensively, favouring the rapid build-up of nematode populations in the soil, with the detection of several species in a wide variety of hosts. The information gathered on the RKN species found in the country is crucial at a local level for farmers and technicians in adopting sustainable management practices, and in a broader context for decision-makers in establishing phytosanitary measures and monitoring programmes to prevent the introduction and spread of these pests of concern in Europe. This is particularly relevant for quarantine and regulatory purposes.

Elsharkawy et al., Pulavarty et al. and Kim et al. focused on the use of alternative products to control different PPNs, namely through volatiles, microbial fermentation products and bacterial filtrates. The study of terpenes as natural products for the control of nematodes has increased in recent years (Faria et al., 2023). Elsharkawy et al. obtained promising results using monoterpenes as plant-derived natural compounds with nematicidal activity against *M. incognita* under laboratory, greenhouse, and field conditions. Carvone, cuminaldehyde, cineole, and linalool were effective for the control of root-knot nematode of tomato. Pulavarty et al. tested microbial fermentation products which are organic based soil health products, against the golden potato cyst nematode (PCN), *Globodera rostochiensis*, a major threat to potato crop in many countries (Price et al., 2021). These formulations provided by Alltech displayed nematicidal properties against PCN, with no detrimental effects on other soil nematodes and on plant growth promotion. *Burkholderia* sp. are multifunctional plant growth-promoting rhizobacteria (PGPR) with some species known to have nematicidal activity (Meyer et al., 2000; Liu et al., 2022; Zhang et al., 2022). Kim et al. studied the nematicidal effect of *Burkholderia* sp. JB-2 strain in suppressing *M. incognita* on tomato, with promising results. The strain also effectively promoted the growth of tomato plants, in addition to upregulating the gene expression linked to plant defence and growth.

Targeting of major nematode parasitism genes *via* Host Delivered-RNAi (HD-RNAi) to confer silencing is one of the most effective new approaches to limit nematode infection. In the study of Joshi et al., through silencing of the nematode effector gene Mi-msp2 in *Arabidopsis* HD-RNAi lines, a decrease of *M. incognita* infection in CaMV35S:: Mi-msp2-RNAi and pAt2g18140::Mi-msp2-RNAi lines was observed. The tissue-specific HD-RNAi suppression technique has also proven to be a useful tool for the production of transgenic crops. Westerdahl et al. performed field studies involving Easter lilies, a staple of the floral industry, engineered with a rice cystatin gene for migratory nematode resistance, namely to the root-lesion nematode *Pratylenchus penetrans.* The authors concluded that transformed lines although not fully resistant, when planted in the field demonstrated and maintained a degree of resistance to *P. penetrans* and displayed desirable growth and quality characteristics similar to non-transgenic lilies.

In conclusion, the adoption of safer and more selective PPN control strategies, such as bionematicides, biocontrol agents, cultural methods, and plant resistance is proving to be a sound alternative to the sole reliance on synthetic nematicides. Also, the access to DNA sequencing and to genome-editing tools, and the possibility to design new plant incorporated protectants are also paving the way for new possibilities for PPNs control. However, the integration of these control strategies is not always straightforward, and their effectiveness can vary depending on the specific nematode species and the local environment. The rhizosphere is a complex system that contains thousands of microbial species, and their interactions with PPNs are still not fully understood. Further research is needed to better understand the interactions between PPNs, microbial antagonists, and plant resistance mechanisms in order to develop more effective and sustainable control strategies.

## Author contributions

MI: Conceptualization, Writing – original draft. JF: Writing – review & editing. SH: Writing – review & editing.
